# Patients' perceived lack of goal clarity in psychological treatments: Scale development and negative correlates

**DOI:** 10.1002/cpp.2479

**Published:** 2020-06-01

**Authors:** Naline Geurtzen, Ger P.J. Keijsers, Johan C. Karremans, Bea G. Tiemens, Giel J.M. Hutschemaekers

**Affiliations:** ^1^ Behavioural Science Institute Radboud University Nijmegen The Netherlands; ^2^ Pro Persona Research Renkum The Netherlands; ^3^ Department of Clinical Psychological Science Maastricht University Maastricht The Netherlands; ^4^ Indigo Utrecht The Netherlands

**Keywords:** care dependency, goal clarity, symptom severity, therapeutic alliance, treatment goals

## Abstract

Goal setting in psychological treatments may have favourable effects on patients' motivation and treatment outcomes. Therefore, it seems important to detect when patients do not perceive clear treatment goals. The current study presents a questionnaire measuring patients' perceived lack of goal clarity. The cross‐sectional study consisted of 742 adult outpatients with diverse mental disorders. Patients completed the perceived lack of goal clarity questionnaire, and additional items measuring goal setting and evaluation, therapeutic alliance, symptom levels, patients' dependency on their treatment, and their expected and needed number of future treatment sessions. Exploratory factor analysis and reliability analyses resulted in a unidimensional and reliable questionnaire (nine items, *α* = .85). Additional findings showed that 23% of the treatments lacked initial goal setting according to the patients. Also, perceived lack of goal clarity was lower when treatment goals were established explicitly at the start of treatment, were formulated together with the therapist, and were discussed regularly during treatment, and treatment progress was monitored regularly. Moreover, patients reporting their goals as unclear also reported a poorer quality of the therapeutic alliance, higher symptom levels, increased need for future sessions, but also lower levels of care dependency. These findings underscore the importance of perceived goal clarity in psychological treatments, although the relation with actual goal setting remains uncertain.

**Key Practitioner Message**

- A new, brief (nine items) instrument measuring whether patients feel that clear treatment goals are lacking is developed and tested in a large patient sample.
- Almost 25% of the patients report that initial goal setting was lacking, and 30% of the remaining patients report that their treatment goals were addressed only sometimes during treatment.
- Perceived treatment goals are clearer when treatment goals were established at the start of treatment, formulated together with the therapist, discussed regularly during treatment, and when treatment progress was monitored regularly.
- Unclear treatment goals were related to more severe symptoms, more need for additional sessions, and a less satisfying therapeutic alliance.

## 1 INTRODUCTION

Goal setting has been the subject of many studies in various fields of psychology, revealing that goal setting is crucial in the initiation and persistence of behaviour (Locke & Latham, 2002). Not surprisingly, goal setting is also important in psychological treatments, as it was found to have direct favourable effects on patients' distress and well‐being, as well as on their motivation and treatment outcomes (Hart, 1978; Locke & Latham, 2002; Michalak & Grosse Holtforth, 2006; Michalak, Klappheck, & Kosfelder, 2004; Tryon & Winograd, 2011). For example, a randomized controlled trial by Hart (1978) showed that patients who weekly set and evaluated their treatment goals together with their therapist improved significantly more than patients without this extra focus on goal setting and evaluation.

Recent studies elaborated on the issue by arguing that goal setting may benefit the treatment process by facilitating the initial phase of the treatment, guiding the treatment plan, helping to stay focused during the course of treatment, and helping to evaluate the treatment (Michalak & Grosse Holtforth, 2006; Tiemens, Reijs, Van Sonsbeek, & Hutschemaekers, 2010). Also, goal setting helps to determine whether patients and therapists actually share the same goals (i.e., goal consensus), which is relevant as treatment goals do not only imply symptom reduction but may also cover other areas in life, such as an increased well‐being, personal growth, or improvement in the quality of interpersonal relations (Grosse Holtforth & Grawe, 2002; Michalak & Grosse Holtforth, 2006; Pueschel, Schulte, & Michalak, 2011). Goal consensus is seen as a crucial step in building a collaborative bond between patients and therapists (Bordin, 1979; Tryon & Winograd, 2011). As both goal consensus and the quality of the collaborative relationship predict treatment outcomes (Flückiger, Del Re, Wampold, & Horvath, 2018; Lambert & Barley, 2001; Tryon & Winograd, 2011), these findings suggest that clear goal setting may lead to a better therapeutic alliance and, in turn, to better treatment outcomes as well.

An important prerequisite for the potential positive effects of goal setting seems to be that the treatment goal should be perceived as clear by the patient. Indeed, it has been argued that treatment goals should be concrete and specific (Michalak et al., 2004; Michalak & Grosse Holtforth, 2006; Tiemens et al., 2010). However, it seems that not in all treatments clear treatment goals are formulated (Berking, Grosse Holtforth, Jacobi, & Kröner‐Herwig, 2005). In an explorative study, Hellenbrand, Tiemens, and Appel (2007) asked therapists to select some of their ongoing treatments of which they felt that these were continuing for a (too) long period of time. Results showed that almost all of the selected 27 treatments were characterized by the use of vague, non‐specific treatment goals. Although these data do not clarify whether these treatments were lacking clear goals at the start of treatment, or whether goal clarity diminished over the course of treatment, it does suggest that a lack of clear treatment goals is related to prolonged treatment duration. A possible explanation for this relationship is that a lack of (perceived) goal clarity in treatment may increase patients' dependency on the therapist (Geurtzen, Keijsers, Karremans, & Hutschemaekers, 2018). Care dependency refers to patients' submissive stance in treatment, their wish to be close with their therapist, and patients' perception that only that particular treatment could help them dealing with their symptoms (Geurtzen et al., 2018). When treatment goals are perceived as unclear, it might be more difficult for patients to engage proactive in treatment, and patients may have to rely more on the direction of their therapist. Ultimately, this may lead to treatments continuing longer than necessary.

Other reasons for treatment goals being perceived as unclear by the patient may be frequent shifts in treatment goals, as studies found that repeated changes of the initial treatment goals throughout the course of treatment were negatively correlated with treatment outcome (Schulte & Eifert, 2002; Schulte‐Bahrenberg & Schulte, 1993). Treatment goals might be changed when progress is lacking. However, it has been suggested also that therapists change their treatment strategy too soon, too often, and sometimes for the wrong reasons (Schulte & Eifert, 2002). Waller (2009) and colleagues argue that frequent switches of goals, or the lack of clear treatment goals, may lead to treatments drifting away from the initial aims of treatment, resulting in a shift from “doing therapies” to “talking therapies,” also referred to as therapist drift (Schulte & Eifert, 2002; Waller & Turner, 2016). In line, we suppose that frequent changes in goals may lead to goals becoming unclear, which undermines the favourable effects of goal setting on patients' motivation and treatment outcome (see, e.g., Hart, 1978; Michalak et al., 2004; Michalak & Grosse Holtforth, 2006).

Of course, therapists “non‐adherence to the treatment goals” may also reflect flexible and responsive reactions of the therapist to the needs of individual patients, leading to better outcomes in psychotherapy (Owen & Hilsenroth, 2014), for instance, when a first goal has been achieved or when new information or insights places the patients' problems in a different light (Lambert, Harmon, Slade, Whipple, & Hawkins, 2005; Schulte & Eifert, 2002). Additionally, different therapeutic orientations may approach goal setting in different ways. Nevertheless, when treatment goals are strived for, it is relevant to know whether these treatment goals are perceived as clear by the patient, in order to profit from the favourable effects of goal setting.

Hence, the primary aim of the current study is to develop a short and reliable self‐report questionnaire that can be used to detect patients' perceived lack of goal clarity in psychological treatments. There is an extra reason for measuring the patients' perception of goal clarity directly instead of developing a therapist‐rated instrument: It is unclear whether therapists are able to accurately rate their patients' perceived goal clarity. To illustrate, the process of initial goal setting in treatment often lacks explicit goal agreement between patients and therapists (Oddli, McLeod, Reichelt, & Rønnestad, 2014). Also, discrepancies are found between patients and therapists ratings regarding their goal congruence, and goal attainment (Hunsley, Aubry, Verstervelt, & Vito, 1999; Tryon, Blackwell, & Hammel, 2007). Next to the primary aim to develop the relevant questionnaire, the second aim is to investigate whether patients' *perceived* lack of goal clarity relates to patients reports of actual goal setting and monitoring in treatment and whether perceived lack of goal clarity relates to unfavourable treatment factors, such as patients' higher symptom levels, a worse therapeutic alliance, higher levels of care dependency in patients, and potentially longer treatments. We test these correlations in order to obtain a better understanding of the potential reasons and consequences of a perceived lack of goal clarity in treatment practice.

The present paper first describes the development of the self‐report questionnaire and reports its psychometric qualities, found in a large cross‐sectional sample of 742 patients receiving outpatient psychological treatment for a variety of mental health disorders in different programmes. Then, it describes whether, according to the patients, treatment goals were actually established at the start of treatment, whether goals were formulated together with the therapist, regularly addressed during treatment, and whether treatment progress was monitored. Next, we check whether patients' perceived lack of goal clarity is indeed lower when clear goals were formulated in the initial treatment phase, when treatment goals were mutually formulated and agreed upon, when treatment goals and goals attainments were regularly addressed during the course of the treatments, and when progress was monitored during treatment (i.e., concurrent validity). Last, we study correlates of patients' perceived lack of goal clarity by examining its associations with the quality of the therapeutic alliance (including goal consensus), patients' symptom levels, patients' care dependency on their treatments, and patients' estimates of the number of expected future treatment sessions and the number of future treatment sessions needed. We hypothesize that a higher perceived lack of goal clarity is related to a lower quality of the therapeutic alliance, higher symptom levels, higher levels of patients' care dependency, and to higher numbers of expected and needed future treatment sessions.

## 2 METHOD

The current study was part of a larger cross‐sectional study on the development of the Care Dependency Questionnaire (CDQ) among patients in mental health care (Geurtzen et al., 2018). We refer to Geurtzen et al. (2018) for a detailed overview of the study.

### 2.1 Participants and procedure

Participants were 742 adult patients with an outpatient treatment in one of the Generalistic Mental Health Care (GMHC) programmes or Specialized Mental Health Care (SMHC) programmes of Pro Persona, a large mental health care institution in the Netherlands. The GMHC offered mental health care to patients in a primary care setting and is characterized by short‐term, often time‐limited treatments with a strong focus on increasing patients' self‐management skills. SMHC offered mental health care for patients with long‐term and complex psychological symptoms or with multiple mental health disorders. Treatments in SMHC usually take longer.

At the start of the study, 3,552 patients were selected because they were currently receiving an outpatient treatment in one of the four participating care programmes of Pro Persona (i.e., GMHC, SMHC‐mood disorders, SMHC‐anxiety disorders, or SMHC‐personality disorders). Of the 3,552 patients, 2,949 could be approached digitally via “NETQROM,” a software program used within Pro Persona for routine outcome monitoring purposes. These patients received a digital information letter and informed consent form. In total, 772 patients (26.2% of the 2,949 patients) agreed to participate in the study. At the time of the data collection, response rates on the digitally administered routine outcome monitoring measures were generally quite low in all care programmes (see also Geurtzen et al., 2018). We did not have any reason to believe that attrition in the current study was systematic. Next, 30 patients were excluded because they were still on the waiting list, or because they were minors (<18 years old). Participants received a digital link via “NETQROM” to complete the set of questionnaires. It took them approximately 30 min to complete the measures. These data were combined with other questionnaires administered due to routine outcome monitoring, as well as data derived from electronic patient files, including, for example, the number of treatment sessions patients had received so far.

The final sample consisted of 742 patients, of which 64.6% were females. Patients were on average 41.1 years old (*SD* = 11.0). About half of the patients (48.6%) lived together with a partner (with or without children), and 69.7% had finished secondary education at a lower or intermediate level, whereas 24.3% had finished higher vocational or academic education. Forty‐one percent of the patients reported to have a paid job. Mental health care characteristics were as follows: Eighty‐six patients (11.6%) were treated in outpatient GMHC. The remaining 656 patients (88.4%) were treated in one of the three outpatient SMHC programmes according to the primary diagnosis (mood disorder: *n* = 180, 24.3%; anxiety disorders: *n* = 127, 17.3%; and personality disorders: *n* = 348, 46.9%). Most patients within GMHC and SMHC received individual (or individual combined with group) psychological treatment (e.g., cognitive therapy, exposure therapy, behaviour activation, schema‐focused therapy, or eye movement desensitization and reprocessing). A minority, 33.3% of all patients, received a combination of psychological treatment and pharmacotherapy. Finally, there was a huge variety in treatment duration at the time of the current study: On average, patients received 174 treatment sessions (face‐to‐face sessions, e‐health, etc., *SD* = 352.82) at the moment that they participated in the current study. However, 25% of the patients have completed only up to 15 sessions, 50% of all patients have completed up to 50 sessions so far (i.e., median), and 75% of all participants have completed up to 173.5 sessions at the time that the study took place.

### 2.2 Measures

#### 2.2.1 Lack of goal clarity

Based on face validity, 13 items measuring patients' perceived lack of goal clarity were formulated. For example, “It is clear to me what the focus of my treatment is”; “I find it difficult to indicate what the desired result of my treatment is”; or “The goal of the treatment tends to change from time to time.” The items of the current scale measuring patients' lack of goal clarity were originally developed as part of the CDQ. Lack of goal clarity was removed from the CDQ, however, because it was eventually argued to be a potential predictor of care dependency rather than an aspect of the concept care dependency itself (see footnote in Geurtzen et al., 2018).

All items were rated on a 7‐point Likert scale, ranging from 1 (*completely disagree*) to 7 (*completely agree*). The scores of positively formulated items were pooled so that higher scores of all items reflect a greater lack goal clarity as perceived by the patients. After an initial round of patients' feedback in a pilot study among 36 adults of academic outpatient treatment centre, two unclearly formulated items were removed, resulting in an 11‐item version of the current questionnaire. The results section reviews the relevant psychometric analyses, and Table 1 presents the original 11‐item questionnaire. Table 3 presents Cronbach's alphas of the final nine‐item version of the questionnaire, as well as the Cronbach's alphas of the other study measures described below.

**TABLE 1:** Item statistics and factor loadings of lack of goal clarity scale

#	Item			Factor loadings^a^			
		*M*	*SD*	1	2	*r*_it_ ^b^	Cronbach's α if item deleted
1	[4] It is clear to me what the focus of my treatment is^c^	2.88	1.52	**.68**	−.18	.62	.76
2	[7] I have the feeling there is no clear purpose in my treatment	3.06	1.75	**.73**	−.13	.65	.75
3	[12] I find it difficult to indicate what the desired result of my treatment is	3.92	1.77	**.58**	.17	.49	.77
4	[16] The goal of the treatment tends to change from time to time	3.71	1.55	**.54**	.29	.44	.77
5	[22] I understand the connecting thread in my treatment^c^	3.06	1.54	**.68**	−.07	.60	.76
6	[25] During conversations with my therapist I'd prefer to discuss everything that is on my mind	5.14	1.46	.06	**.53**	−.04	.82
7	[29] For a long time now my treatment has not focused on the problems for which I came into treatment at the time	3.13	1.64	**.53**	.10	.46	.77
8	[33] The therapist and I follow a predefined plan in the treatment^c^	3.46	1.50	**.51**	−.15	.47	.77
9	[37] It is unclear to me what I would like to have achieved exactly at the end of the treatment	3.43	1.75	**.57**	.09	.49	.77
10	[41] What we discuss during the treatment session depends on what occupies me the most at that moment	5.33	1.23	.05	**.44**	−.02	.81
11	[45] It is more and more unclear to me what the focus of my treatment is	3.10	1.61	**.81**	.00	.71	.74

*Note*: *N* = 742. The original questionnaire in the current study was in Dutch. The English adaptation of the items in the table is based on a “forward‐and‐backward” translation procedure by a professional translation office including a native English speaker. In addition, the items in the current study were part of the original 54‐item questionnaire measuring patients' care dependency, see footnote in Geurtzen et al. (2018). The numbers between brackets show the original numbering of these items. All items were rated on a 1‐ to 7‐point Likert scales from 1 (*completely disagree*) to 7 (*completely agree*).

^a^ Factor loadings based on Principal Axis Factoring with Promax rotation. Factor loadings over .32 appear in boldface. The first factor explained 37.8% of the variance, and the second factor explained 12.2% of the variance.

^b^ *r*_it_ = part‐whole corrected item‐total correlation.

^c^ Item reversed scored.

#### 2.2.2 Goal setting and evaluation

The following four additional items were administered to measure the degree of goal setting and evaluation in treatment: (1) “At the start of the treatment, were one or more treatment goals set?” (1 = *yes*; 2 = *no*); if yes, (2) “Did you set this treatment goal or these treatment goals together with your therapist?” (1 = *yes*; 2 = *no*); (3) “Are your treatment goal(s) discussed during the therapy sessions?” (1 = *Yes*, *at* [*almost*] *each session*; 2 = *often*; 3 = *sometimes*; 4 = [*almost*] *never*); and (4) “Is your progress measured during the treatment? Consider the completion of questionnaire(s) or having an evaluation discussion, etc.” (1 = *Yes*, *at* [*almost*] *each session*; 2 = *often*; 3 = *sometimes*; 4 = [*almost*] *never*).

#### 2.2.3 Therapeutic alliance

The therapeutic alliance was measured with the Dutch adaptation of the short Working Alliance Inventory for patients (WAI‐12). The original 36‐item English version of the WAI was developed by Horvath and Greenberg (1989) and translated into Dutch by Vertommen and Vervaeke (1990). The short English version of the WAI (WAI‐SR) was developed by Hatcher and Gillaspy (2006), and the adapted Dutch version based on Vertommen's and Vervaeke's translation was developed by Stinckens, Ulburghs, and Claes (2009). The Dutch short version of the WAI, WAI‐12, has been shown to have sufficient validity and reliability (Smits, Luyckx, Smits, Stinckens, & Claes, 2015; Stinckens et al., 2009) and measures the degree of agreement between patients and therapist with regard to the treatment *tasks* and the treatment *goals* (i.e., goal consensus), and the strength of the *bond* between patients and therapists, all perceived by the patient (e.g., “My therapist and I are working towards mutually agreed upon goals,” or “My therapist and I respect each other”). All items were rated on a 5‐point Likert scale, ranging from 1 (*rarely or never*) to 5 (*always*), and higher scores reflect a better therapeutic alliance.

#### 2.2.4 Psychological symptoms and functioning

Patients' levels of symptoms and functioning were measures with the Dutch adaptation of the Outcome Questionnaire (OQ‐45.2, Lambert et al., 1996; Dutch adaptation by de Jong, Nugter, Lambert, & Burlingame, 2009). The OQ‐45.2 consists of 45 items measuring symptomatic distress, difficulties in interpersonal relationships, and problems regarding functioning in the social role (e.g., “I feel weak,” or “I am not working/studying as well as I used to”). Items were rated on a 5‐point Likert scale, ranging from 0 (*never*) to 4 (*almost always*), and a higher score reflects more symptoms or problems in functioning. The Dutch adaptation has acceptable psychometric properties (de Jong & Nugter, 2004).

#### 2.2.5 Care dependency

Patients' care dependency levels were measured with the CDQ (Geurtzen et al., 2018). This questionnaire contains 18 items covering three dimensions of care dependency; patients' submissive stance in treatment (e.g., “I present all my decisions to my therapist”), patients' need for contact with the therapist (e.g., “I dread ending the contact with my therapist at the end of the treatment”), and the lack of perceived alternatives (e.g., “In my opinion, this treatment is the only way of ridding myself of my complaints”). All items were answered on a 7‐point Likert scale, ranging from 1 (*completely disagree*) to 7 (*fully agree*), and higher scores indicate higher levels of care dependency. The scale was shown to have adequate psychometric characteristics (Geurtzen et al., 2018).

#### 2.2.6 Expected and needed number of future treatment sessions

As the design of the current study was cross sectional, patients were in different phases of their treatment. As a result, treatment duration could not be estimated. In order to get some idea of the expected treatment length, we asked patients about their *expected* number of future treatment sessions: “How long do you think that your current treatment will continue? Please report a number reflecting the number of future sessions.” Also, we were interested in their *need* for future treatment sessions. Therefore, we also asked them “How many treatment sessions do you think you still need in order to be able to function independently after treatment?” Again, participants were asked to report a number reflecting the number of needed treatment sessions.

### 2.3 Data analysis

An exploratory factor analysis on the lack of goal clarity scale was performed to examine whether all 11 items loaded on one underlying factor or not (i.e., Principal Axis Factoring with Promax rotation). To determine the internal consistency, Cronbach's alpha was calculated. Next, patients' responses on the four items measuring goal setting and evaluation in treatment were examined by means of the descriptive frequencies: (1) initial goal setting (yes/no); (2) goal setting together with their therapist (yes/no); (3) bringing up the treatment goals during treatment (yes [almost] every session/often/sometimes/[almost] never); and (4) evaluation of treatment progress: (yes [almost] every session/often/sometimes/[almost] never).

Before examining whether patients' perceived lack of goal directedness differed between the degree of goal setting and evaluation based on the previous four questions, we first checked whether there were any differences between patients from different care programmes (GMHC vs. SMHC) by means of a general linear model (GLM) univariate test. Then, multiple GLM univariate tests were conducted to check whether the final scale scores measuring patients' lack of goal clarity (dependent variable) differed for patients responding differently on the four items measuring goal setting and evaluation, whilst controlling for treatment duration by adding this variable as a covariate in the model.

Subsequently, partial Pearson and partial Spearman's rho correlations were examined to determine the associations between the lack of goal clarity and the study variables, namely, the therapeutic alliance, symptom levels, patients' care dependency (i.e., patients' submissive stance, patients' need for contact with their therapist, and patients' perceived lack of alternative options), and patients' expected and needed number of future treatment sessions, whilst controlling for treatment duration so far.

## 3 RESULTS

Exploratory factor analysis yielded two underlying factors: the first factor explaining 37.8% of the variance and the second one 12.2% (see Table 1). Factor loadings showed that all items loaded high on the first factor, except for two items (i.e., Item 6 “During conversations with my therapist I'd prefer to discuss everything that is on my mind,” and Item 10 “What we discuss during the treatment session depends on what occupies me the most at that moment,” see Table 1). The two factors correlated negatively (*r* = −.29). Cronbach's alpha on the initial 11 items was .79 but increased to .85 when Items 6 and 10 were excluded from the calculation. The second factor, seeming to address the degree of “tuning in with the patient” or “following the patient,” consisted of only two items (i.e., Items 6 and 10) but failed to result in an internally consistent subscale (Cronbach's *α* = .43). The final scale thus consisted of the nine remaining items with high loadings on the first factor, measuring patients' lack of goal clarity in treatment. Table 1 shows an overview of the items statistics and factor loadings per item.

Descriptive statistics of the four items measuring goal setting and evaluation in treatment (see Table 2) revealed that 77% of all patients' reported that treatment goals were established at the start of their treatment, meaning that almost one out of four treatments (23%) lacked initial goal setting according to patients' responses. When treatment goals were established according to the patients, they reported that these goals were almost always formulated together with their therapist (91%). Also, more than 60% of the patients who reported that initial treatment goals were established stated that these treatment goals were discussed (almost) always (29%) or often (32.3%) during treatment. Thirty percent of the patients on the other hand reported that treatment goals were addressed only sometimes during treatment, and 8.7% reported that their treatment goals were (almost) never discussed during treatment at all. Last, of all participants, almost 60% reported that their treatment progress was monitored (almost) always during treatment (20.1%) or often (38.3%). On the other hand, 32% of the patients reported that their progress was monitored only sometimes during treatment, and 9.6% reported that their progress was (almost) never monitored at all.

**TABLE 2:** Frequencies of the four items measuring goal setting and evaluation, means of patients' perceived lack of goal clarity, and results of the GLM univariate tests per item

Item	Response options	*n*	*M*	95% CI	*F*	*p*	*η*_*p*_^2^
1. At the start of the treatment, were one or more treatment goals set?	Yes	570	3.13	[3.04, 3.22]			
	No	171	3.91	[3.75, 4.08]			
	Total	741	3.31		64.92	<.001	*η*_*p*_^2^ = .09
2. Did you set this treatment goal or these treatment goals together with your therapist?	Yes	520	3.09	[3.00, 3.18]			
	No	50	3.68	[3.38, 3.97]			
	Total	570	3.13		14.14	<.001	*η*_*p*_^2^ = .03
3. Are your treatment goal(s) discussed during the therapy sessions?	Yes, at (almost) every session	165	2.83	[2.68, 2.99]			
	Often	184	2.91	[2.77, 3.06]			
	Sometimes	171	3.34	[3.19, 3.49]			
	(Almost) never	50	4.32	[4.04, 4.59]			
	Total	570	3.14		34.39	<.001	*η*_*p*_^2^ = .17
4. Is your progress measured during the treatment? Consider the completion of questionnaire(s) or having an evaluation discussion, and so on.	Yes, at (almost) every session	149	2.95	[2.77, 3.12]			
	Often	284	3.05	[2.92, 3.18]			
	Sometimes	237	3.63	[3.49, 3.76]			
	(Almost) never	71	4.06	[3.81, 4.32]			
	Total	742	3.31		28.64	<.001	*η*_*p*_^2^ = .12

*Note*: The potential range of patients' perceived lack of goal clarity is from 1–7. *M* = estimated marginal means based on the GLM univariate tests, controlled for the number of treatment sessions patients completed so far.

Abbreviations: CI, confidence interval; GLM, general linear model; *η*_*p*_^2^, partial eta^2^.

Results showed no significant difference between patients from the GMHC and SMHC programmes regarding their perceived lack of goal clarity, *F*(1, 740) = 3.27, *p* = .071. As we were actually somewhat surprised by this null finding, we further explored potential differences between the four care programmes separately. A GLM univariate variance analysis showed a weak but significant difference between the four care programmes with regard to patients' lack of goal clarity, *F*(3, 738) = 20.87, *p* < .001, *η*_*p*_^2^ = .08. Post hoc analyses revealed that the lack of goal clarity was significantly lowest in the SMHC‐anxiety disorders (*M* = 2.69), followed by GMHC (*M* = 3.12), and significantly highest in both the SMHC‐personality disorders (*M* = 3.49) and SMHC‐mood disorders (*M* = 3.48). However, adding treatment programme with the four separate groups in the subsequent analyses (i.e., as an extra fixed factor in the GLM univariate tests as presented in Table 2, or as an extra control variable in the partial correlations as presented in Table 3), did not result in any significant changes in the results of these analyses.

**TABLE 3:** (Sub)scale statistics, Cronbach's alpha, and partial Pearson or Spearman's rho correlations between study variables

(sub)Scale	*M* (*SD*)	Range	*α*	2	3	4	5^a^	6	7	8	9^b^	10^b^
1. Lack of goal clarity	3.31 (1.09)	1.00–6.78	.85	−.66^***^	−.66^***^	−.44^***^	.31^***^	−.16^***^	−.14^***^	−.17^***^	.08	.14^***^
2. Therapeutic alliance: agreement tasks	3.16 (0.97)	1.00–5.00	.89	—	.87^***^	.66^***^	−.31^***^	.31^***^	.28^***^	.29^***^	−.03	−.09^*^
3. Therapeutic alliance: agreement goals	3.20 (0.95)	1.00–5.00	.87		—	.70^***^	−.29^***^	.29^***^	.27^***^	.25^***^	−.06	−.09^*^
4. Therapeutic alliance: bond	3.19 (0.94)	1.00–5.00	.86			—	−.28^***^	.23^***^	.29^***^	.20^***^	−.06	−.08^*^
5. Symptom levels and functioning^a^	1.85 (0.56)	0.11–3.38	.94				—	.12^*^	.16^**^	.25^***^	.24^***^	.35^***^
6. Care dependency: submissive stance	4.05 (1.08)	1.00–6.80	.74					—	.53^**^	.66^**^	.08^*^	.16^***^
7. Care dependency: need for contact	4.31 (1.39)	1.00–7.00	.81						—	.65^***^	.16^***^	.27^***^
8. Care dependency: lack alternatives	4.19 (1.10)	1.00–7.00	.86							—	.23^***^	.32^***^
9. Expected number of future treatment sessions^b^ ^,^ ^c^	58.83 (171.34)	0–1,000	—								—	.77^***^
10. Needed number of future treatment sessions^b^ ^,^ ^d^	80.42 (204.13)	0–1,000	—									—

*Note*: *N* = 665. All Pearson and Spearman's rho correlations are controlled for the number of treatment sessions patients completed so far.

^a^ *n* = 338, because only questionnaires were selected that were completed within 4 weeks before or after the administration of the other questionnaires in the current study.

^b^ Spearman's rho correlations.

^c^ Skewness = 4.91, kurtosis = 23.65.

^d^ Skewness = 3.92, kurtosis = 14.48.

^*^ *p* < .05.

^**^ *p* < .01.

^***^ *p* < .001.

As there was a significant difference between the GMHC and SMHC‐programmes regarding their average treatment duration (i.e., number of treatment sessions patients had completed at the moment that the present data were collected in treatment), *F*(1, 667) = 12.65, *p* < .001, *η*_*p*_^2^ = .019, we checked whether treatment duration and patients' lack of perceived goal clarity correlated significantly. Results showed a small but significant correlation (*r* = .08, *p* = .046), so we did control for treatment duration (i.e., number of treatment sessions so far) in the subsequent analyses.

Next, we examined whether patients' perceived goal clarity related to goal setting and evaluation, controlled for treatment duration. Results showed that patients' perceived lack of goal clarity in treatment, measured with the final nine‐item version of the questionnaire, was significantly lower for those patients who reported that treatment goals were set at the onset of treatment, as compared with patients who reported that no treatment goals were set in the initial phase of treatment (see Table 2). Within the first group, patients who set their treatment goals together with their therapists reported significantly lower levels of the lack of goal clarity as compared with patients who did not set their treatment goals together with their therapist. Also, the lack of goal clarity was significantly larger when initial treatment goals were less frequently discussed during treatment. Furthermore, based on the total sample, the lack of goal clarity was higher when treatment progress was not evaluated on a regular basis.

Next, partial Pearson and Spearman's rho correlations (i.e., controlled for number of treatment sessions so far, see Table 3) showed that the lack of goal clarity was strongly related to the perceived quality of the therapeutic alliance. More specifically, a greater perceived lack of goal clarity was related to lower agreements with the therapists on the treatment tasks and the treatment goals, as well as with a weaker perceived bond with the therapist. In addition, higher levels of the perceived lack of goal clarity were related to higher levels of current psychological symptoms and problems regarding interpersonal relationships and functioning. Further, a greater lack of goal clarity was related to lower levels of care dependency, indicated by a somewhat less submissive stance in treatment, less need for contact with the therapist, and lower perceived lack of alternatives options. Last, patients' perceived lack of goal clarity was significantly but weakly correlated to a larger number of patients' needed number of future treatment sessions, but not to patients' expected number of future sessions.

## 4 DISCUSSION

Results showed that nine of the initial 11 items measuring patients' perceived lack of goal clarity in treatment resulted in a reliable and unidimensional scale. Patients' responses on the additional items measuring goal setting and evaluation in treatment showed that in almost 25% of the treatments, no treatment goals were formulated at the start of the treatment. When treatment goals were established, approximately 40% of the patients reported that these goals were not regularly addressed during treatment. As expected, perceived lack of goal clarity was higher when no treatment goals were set in the initial phase of treatment, when treatment goals were not set together with the therapist (e.g., only by the patient itself), when the treatment goals were not readdressed regularly during treatment, and when treatment progress was not monitored on a regular basis during treatment. These findings suggest that the newly developed scale, perceived lack of goal clarity, seems to measure the underlying concept as was intended (i.e., good concurrent validity).

Correlational analyses have provided further evidence for the potential beneficial effects of clear goal setting. Perhaps most prominent, we found moderate to strong correlations between perceived goal clarity and the perceived quality of the therapeutic alliance. These correlations indicate that in the perception of the patients, a stronger lack of goal clarity is related to a poorer quality of the therapeutic alliance. Interestingly, a lack of perceived goal clarity is related not only to the two subscales of the WAI‐12 that indicate a lower agreement between patient and therapist regarding treatment goals and tasks, considered a crucial part of the therapeutic alliance, but also to the third WAI‐12 subscale, the emotional bond with the therapist. More specifically, lack of goal clarity is related to the patients' impressions that they and their therapists do not really agree on means and goals of treatment, considered as crucial for the therapeutic alliance, and that they were less able to establish a close bond. The relation between perceived goal clarity and the therapeutic alliance is reasonably bidirectional and reinforcing: A better therapeutic alliance may increase (mutual) fine‐tuning of treatment goals, and trying to achieve clear and mutually agreed upon treatment goals may promote mutual trust and understanding. The association between perceived goal clarity and the therapeutic alliance, again, stresses the importance of clear treatment goals, because the quality of the therapeutic alliance in general, as well as goal consensus between patients and therapists in particular, is consistently related to favourable treatment outcomes (e.g., Flückiger et al., 2018; Lambert & Barley, 2001; Tryon & Winograd, 2011).

The latter suggestion is supported by our results, showing that a perceived lack of goal clarity is associated with somewhat higher levels of psychopathology and more difficulties in daily functioning. The correlational analyses in the current sample do not reveal the causal direction of this relationship. Perceiving treatment goals as less clear may result in less effective treatments (e.g., via lack of treatment focus and/or poorer quality of the therapeutic alliance). But it might be possible also that it is more difficult to formulate agreed upon treatment goals in patients with higher symptom levels or that it is harder for them to focus on the treatment goal. A third possibility that cannot be ruled out is that patients who experience more symptoms might be inclined to generally offer negatively biased responses, or vice versa (better treatment progress may lead to the perception of clearer treatment goals). These latter explanations imply that a lack of perceived goal clarity might not be an indicator of treatment quality but of the complexity of the patients' problems. Nevertheless, previous studies suggested an activating role of goal setting in treatment, as well as a positive association between goal pursuing and well‐being (e.g., Locke & Latham, 2002; Michalak & Grosse Holtforth, 2006), meaning that higher levels of psychological symptoms may actually also result from the lack of goal clarity. More research is needed to examine the causal effects of the lack of goal clarity on treatment outcomes, and the underlying mechanisms explaining this relationship.

With regard to the association between patients' perceived lack of goal clarity and patients' dependency on the treatment, we have found, in contrast to our hypothesis, that patients who report unclear goals in treatment are slightly less dependent on the treatment and the therapist, as reflected in a lesser submissive stance, a reduced need for contact with their therapist, and a stronger belief in alternatives options. Thus, our hypothesis that care dependency and the lack of goal clarity go hand in hand has not been confirmed by the present data. The most likely explanation is that goal clarity is associated with a higher satisfaction with the treatment, thereby enhancing the patients' eagerness to further profit from the treatment, including the help of their therapist. Put differently, when patients experience a strong therapeutic alliance, they may feel that they work together with their therapists regarding achieving certain treatment goals, which also includes the perception of patients that they need the help of their therapist (i.e., are dependent on). Future research is needed to confirm and clarify the positive associations between goal clarity and care dependency.

With regard to patients' expected and needed number of future treatment sessions, correlations showed that patients' perceived lack of goal clarity in treatment is related to patients' higher need for more future sessions (but not with the expected number of future sessions). Although the correlation was weak, it fits the idea that patients who perceive unclear treatment goals may also experience less progress in treatment and tend to believe that more treatment sessions are needed, which could possibly relate to prolonged treatment duration (see e.g., Hellenbrand et al., 2007). Again, the uncertainty about the causal direction of this relationship leaves room for other interpretations as well. For example, it is possible that higher levels of patients' symptoms have influenced (“biased”) patients' perceived lack of goal clarity in treatment and their estimated need for future treatment sessions. Future research is needed to confirm that the degree of perceived goal clarity is indeed lower in long‐term treatments and that a lack of goal clarity leads to prolonged treatment durations.

The current study has a couple of strengths, such as the very large naturalistic patient sample covering the most common psychiatric disorders. Also, the collected data are heterogeneous, what makes our findings fairly robust. However, several limitations have to be mentioned also. First, the heterogeneity of the data also implies that confounds may have influenced our findings, which makes it difficult to interpret the results in a conclusive way. For example, participants were approached at a “random” point in time during their treatments, meaning that some patients had only just started their treatment whereas others had been in treatment for years. Although we controlled for treatment duration in our analyses, investigating the relation between lack of goal clarity and final treatment duration was not possible with our current data. Also, the sample covers patients with a variety of primary disorders and a variety of treatment characteristics (e.g., different treatment interventions; different locations and settings; time‐limited vs. time‐unlimited treatments; and individual treatments vs. a combination of group and individual treatments). These aspects may have influenced our results to a certain extent, as it has been not possible to control for all these potential confounds.

A second limitation is that, due to the correlational design of our study, we can only speculate about the causality of our findings, and we have to keep in mind that some of the correlations presented in the current study are only weak to moderate. A third limitation worth mentioning is that we measured the lack of goal clarity by means of patient self‐report only. On the one hand, we have made it clear from the onset of the present study that the patient *perceived* (lack of) goal clarity probably is the most important aspect of goal setting in treatment, when it comes to the motivating and activating role of treatment goals. On the other hand, the ratings of patients are likely to be influenced by other issues, such as patients' symptoms levels, and may thus not necessarily reflect the “objective” presence and clarity of the treatment goals. The patients' perception that no treatment goals have been set at the start of their treatments might also be affected by confounds such as “negativity biases” or “memory constrains” of patients who have started treatment a long time ago.

Future research should try to combine the more or less objective information regarding goal setting derived from patient files with patients' perception and therapists' perception of the goal clarity of the treatment. Also, research should cover multiple therapeutic orientations, as goal setting may not have the same weight in different therapeutic modalities. Perhaps most importantly, additional randomized controlled trials such as Hart's study (1978) are needed in current mental health care practice, to confirm whether goal setting in treatment does indeed lead to better treatment outcomes, and under which conditions. Because one could speculate that under certain circumstances, it can be preferred to work with the patient without the explicit formulation of treatment goals at the start of treatment.

To conclude, the current study presents a short and reliable questionnaire that can be used to spot patients' perceived lack of goal clarity during treatment. The correlational findings correspond with earlier empirical findings that (perceived) clear treatment goals are important in effective treatments. Our hope is that the present research will inspire and help practitioners to assess and monitor patients' perceived goal clarity and will inspire future research to examine this important issue in more depth.
